# Dietary supplementation with probiotics regulates gut microbiota structure and function in Nile tilapia exposed to aluminum

**DOI:** 10.7717/peerj.6963

**Published:** 2019-06-03

**Authors:** Leilei Yu, Nanzhen Qiao, Tianqi Li, Ruipeng Yu, Qixiao Zhai, Fengwei Tian, Jianxin Zhao, Hao Zhang, Wei Chen

**Affiliations:** 1 School of Food Science and Technology, Jiangnan University, Wuxi, China; 2 State Key Laboratory of Food Science and Technology, Jiangnan University, Wuxi, China; 3 International Joint Research Laboratory for Probiotics, Jiangnan University, Wuxi, China; 4 National Engineering Research Center for Functional Food, Jiangnan University, Wuxi, China; 5 (Yangzhou) Institute of Food Biotechnology, Jiangnan University, Yangzhou, China; 6 Beijing Innovation Centre of Food Nutrition and Human Health, Beijing Technology & Business University, Beijing, China

**Keywords:** Probiotic, *Lactobacillus plantarum*, Gut microbiota, Aquaculture, Nile tilapia, Aluminum

## Abstract

**Backgrounds and aims:**

Aluminum contamination of water is becoming increasingly serious and threatens the health status of fish. *Lactobacillus plantarum* CCFM639 was previously shown to be a potential probiotic for alleviation aluminum toxicity in Nile tilapia. Considering the significant role of the gut microbiota on fish health, it seems appropriate to explore the relationships among aluminum exposure, probiotic supplementation, and the gut microbiota in Nile tilapia and to determine whether regulation of the gut microbiota is related to alleviation of aluminum toxicity by a probiotic in Nile tilapia.

**Methods and results:**

The tilapia were assigned into four groups, control, CCFM639 only, aluminum only, and aluminum + CCFM639 groups for an experimental period of 4 weeks. The tilapia in the aluminum only group were grown in water with an aluminum ion concentration of 2.73 mg/L. The final concentration of CCFM639 in the diet was 10^8^ CFU/g. The results show that environmental aluminum exposure reduced the numbers of *L. plantarum* in tilapia feces and altered the gut microbiota. As the predominant bacterial phyla in the gut, the abundances of Bacteroidetes and Proteobacteria in aluminum-exposed fish were significantly elevated and lowered, respectively. At the genus level, fish exposed to aluminum had a significantly lower abundance of *Deefgea*, *Plesiomonas,* and *Pseudomonas* and a greater abundance of *Flavobacterium*, *Enterovibrio*, *Porphyromonadaceae uncultured*, and* Comamonadaceae*. When tilapia were exposed to aluminum, the administration of a probiotic promoted aluminum excretion through the feces and led to a decrease in the abundance of *Comamonadaceae, Enterovibrio* and *Porphyromonadaceae*. Notably, supplementation with a probiotic only greatly decreased the abundance of *Aeromonas* and *Pseudomonas*.

**Conclusion:**

Aluminum exposure altered the diversity of the gut microbiota in Nile tilapia, and probiotic supplementation allowed the recovery of some of the diversity. Therefore, regulation of gut microbiota with a probiotic is a possible mechanism for the alleviation of aluminum toxicity in Nile tilapia.

## Introduction

Aluminum, the third commonest chemical element and the most abundant metal on earth, is ubiquitous in the environment. In recent years, environmental aluminum levels have increased due to diverse anthropogenic activities such as water treatment, eutrophic lakes control, mining operations, and industrial landfill (Fernandez-Davila et al., 2012; Garcia-Medina et al., 2011). The aluminum level reaches 5.7 mg/L in certain rivers and lakes in England, the United States, China, and Brazil (Camargo, Fernandes & Martinez, 2009; Da Cruz et al., 2015), research has been shown that concentration of aluminum ranging from 0.1 to 0.2 mg/L can be harmful to fish (Baker & Schofield, 1982; Wang et al., 2013). Excessive aluminum can accumulate in multiple fish tissues and organs and exert adverse effects on the blood circulation and on endocrine, metabolic and reproductive function (Azmat, Javed & Jabeen, 2012). Excess aluminum ions in water were reported to cause mortalities and decreasing population of *Atlantic salmon* in Norway, southeast Canada, and the northeastern United States (Monette & McCormick, 2008). Aluminum contamination causes economic losses in aquaculture and poses potential human health risks from consumption of aquatic products.

Microorganisms produce a variety of metabolites that can have remarkable effects on the external environment and on the host, including changes in pH, suppression of inflammation, and detoxification (Berdy, 2005; Louis, Hold & Flint, 2014). Hence, the gut microbiota can significantly alter the host’s physiology and its metabolism of nutrients and exogenous toxic substances, and it can shape the microbiome and immune systems (Ni et al., 2014). They may be important mediators of the bioavailability and toxicity of toxic metals. Indeed, long-term toxic metal exposure, including aluminum, lead and chromium, altered the composition of intestinal microbiota (Wu et al., 2017; Zhai et al., 2017).

Lactic acid bacteria probiotics, which are generally derived from humans or food products, are generally recognized as safe (GRAS) strains (Farnworth, 2008) and have been widely applied in various situations, including aquaculture, to improve food safety (Sihag & Sharma, 2012). Some probiotics have been used in the culture of some aquatic organisms to promote growth and control infectious disease. They can improve the host’s intestinal microflora balance and increase the protective effect against pathogenic bacteria (Lahti et al., 2013; Pirarat et al., 2015). For example, *L. johnsonii* La1*, L. rhamnosus* LC705*, B. lactis* Bb12*, L. casei* Shirota and others can effectively control infection with *Vibrio anguillarum*, *Flavobacterium psychrophilum* and *Aeromonas salmonicida,* to prevent furunculosis in rainbow trout (Nikoskelainen et al., 2001), and *L. rhamnosus* GG can control infection of tilapia by *Edwardsiella tarda* and *Streptococcus agalactiae* (Pirarat et al., 2006; Pirarat et al., 2015).

Our previous study showed that supplementation with probiotic *L. plantarum* CCFM639, a strain with superb aluminum binding and tolerance abilities, decreased the aluminum level in tissues and alleviated aluminum toxicity by preventing oxidative stress and histopathological changes in tilapia (Yu et al., 2017). However, it remains unclear whether the mechanisms of recovery and alleviation are related to the gut microbiota. Therefore, we investigated alterations in the composition and structure of the intestinal microbiota in tilapia after aluminum exposure and the addition *L. plantarum* CCFM639 to their daily feed.

Tilapia is one of the most important aquatic species in aquaculture worldwide and is farmed in more than 120 countries and territories (Junning et al., 2018). In 2015, its production accounted for more than 10% of all farmed fish worldwide (FAO, 2015). Moreover, tilapia are recognized as a good biological model because they are easy to handle, culture, and maintain in the laboratory (Korkmaz et al., 2009), and because they display excellent stress sensitivity (Zheng et al., 2016). The aim of the study was to explore whether the regulation of gut microbiota is an aluminum toxicity alleviation mechanism exerted by probiotics in tilapia.

## Materials and Methods

### Probiotic and fish diet preparation

*L. plantarum* CCFM639, kindly provided by the in-house Culture Collections of Food Microbiology (CCFM), Jiangnan University (Wuxi, China), was inoculated in MRS broth (Qingdao Hopebio, China) and in a static condition at 37 °C for 18 h. After centrifugation at 8,000 g at 4 °C for 5 min, the medium was removed and the cell pellets were suspended with sterile normal saline solution(0.85%) to one-hundredth of the original medium volume. The pellets were mixed with the fish basal diet using a sterile spreader to distribute the bacterial cells evenly and to achieve a final probiotic concentration of 10^8^ CFU/g in the feed. The formula and nutrient levels of the basal fish diet were consistent with those in previous studies (Yu et al., 2017). The dose of the *L. plantarum* strain was selected on the basis of previous reports (Heo et al., 2013; Ridha & Azad, 2012; Ridha & Azad, 2016). The bacterial concentration in the fish diet was also confirmed by colony counting. The bacteria-containing feed was prepared weekly and stored at 4 °C before use.

### Experimental design

One hundred ninety-two male tilapia were purchased from the Freshwater Fisheries Research Centre of the Chinese Academy of Fishery Sciences in Wuxi and stocked in a cylindrical aquarium (0.6 m^2^ × 0.85 m) at a fish loading ratio of 1.09 g/L for 3 weeks. The average (±SEM) weight of the tilapia was 34.01 ± 0.19 g. The amount of feed consumed each day was 3% of the average body weight of the fish. The fish were fed manually at 9 am and 5 pm each day. After a 3-week adaptation period, the fish were fasted for 1 day. The four groups are listed in Table 1, including the control group, the probiotic group (639 only), the aluminum exposure group (Al only) and the probiotic intervention group (Al + 639) randomly. The fish in each group were randomly assigned to three tanks with 16 tilapias in each tank. In the aluminum exposure group, the tilapia were grown in water with an aluminum ion concentration (AlCl_3_•6H_2_O) of 2.73 mg/L. The selection of this dose was based mainly on the aluminum exposure doses reported in drinking water, rivers and lakes in previous studies (Yu et al., 2017). The test period was 4 weeks, the freshwater was changed and the aluminum level in the water was checked every 2 days. Inductively coupled plasma mass spectrometry (ICP-MS; NexIon-300X; PerkinElmer) was used to determine the amount of aluminum in the water.

**Table 1:** Experimental groups of tilapia with and without aluminum exposure and probiotic feed.

Group	Experiment time (4 weeks)
Control	Basic feed + normal water
639 only	probiotic feed + normal water
Al only	Basic feed + aluminum water
Al + 639	probiotic feed + aluminum water

**Notes.**

CCFM639 feed, feed containing *L. plantarum* CCFM639 at a concentration of 10^8^ CFU/g; aluminum water, an aqueous environment containing 2.73 mg/L of aluminum ions.

To ensure that fecal samples were not affected by the high level of waterborne aluminum, they were collected quickly after the water was changed to normal water (without aluminum). After collection of the fecal samples, the aluminum ions were added into the water. At the end of the assay, the tilapia were sacrificed under anesthesia after 24 h of fasting. The entire intestinal tract was removed under aseptic conditions and 0.2 g of intestinal contents was squeezed and collected in sterile tubes for analysis of the intestinal microbiota.

The animal experiments was approved by the Ethics Committee of Jiangnan University, China (JN No. 20151027-1129-3), and all procedures about the care and use of experimental animals followed the guidelines set by the European Community (directive 2010/63/EU).

### Determination of fecal aluminum levels

One gram sample of feces was transferred to a microwave digestion tank (OMNI; CEM, UK) with 70% concentrated nitric acid. The microwave digestion system (MARS; CEM, UK) was used. The heating procedure of digestion included three stages: stage 1—power 2000 W, ramp 3:00, temperature 120 °C, hold 3:00; stage 2—power 2000 W, ramp 3:00, temperature 150 °C, hold 10:00; stage 3—power 2000 W, ramp 5:00, temperature 190 °C, hold 16:00. After the temperature fell below 50 °C, the samples were removed and diluted to 50 mL with deionized water. ICP-MS (NexIon-300X; PerkinElmer) was used to determine the amount of aluminum in the samples (Ciavardelli et al., 2012).

### RT-qPCR analyses for mRNA expression of *L. plantarum* in feces

The 0.2 g of fecal samples were collected to extract the total genomic DNA following the instructions of the FastDNA Spin Kit (MP Biomedicals, Santa Ana, CA, USA). The fecal genomic DNA was used as a template, and real-time quantitative polymerase chain reaction (RT-qPCR; CFX96; Bio-Rad, Hercules, CA, USA) was carried out using the specific primer. The primer sequences (5′–3′) referred to previous study (Wang et al., 2018) and as follows, LP-F GGAGCCGCTATTAGTATTTTCAT and LP-R AATACAAGCAAGTCTT185GGACCAG. The Ct value of the fecal sample fluorescence quantification was brought into the corresponding standard curve to calculate the copy number, which was converted into the amount per gram of feces. The standard curve of RT-qPCR was as follows: Ct = −3.1434*lg (copies) + 36.977 (*R*^2^ = 0.9913) (Wang et al., 2018).

### Analysis of gut microbiota

DNA was extracted from 0.2 g of the contents of the whole intestinal tract using an EZNA DNA Kit (Omega Bio-tek, Georgia, USA) and stored at −20 °C. PCR amplification of the 16S rRNA gene was conducted using the forward primer 515F (5′-barcode-GTGCCAGCMGCCGCGG–3′) and the reverse primer 907R (5′-CCGTCAATTCMTTTRAGTTT–3′) (Zhai et al., 2016). Different samples were distinguished with an 8-base barcode and sequenced using a miseq sequencer (Illumina, Inc., California, USA; illumina miseq PE250). The raw data were quality-filtered and aligned using Trimmomatic and FLASH. The operational taxonomic units (OTUs) were clustered with a 97% similarity cutoff using UPARSE (http://drive5.com/uparse/). The chimeric sequences were identified and removed using UCHIME (http://www.drive5.com/uchime/). The OTU germline type was identified by the RDP classifier (http://rdp.cme.msu.edu/) against the SILVA (SSU115) 16S rRNA database using a confidence threshold of 70% (Gajardo et al., 2016; Huyben et al., 2018).

### Data analysis

The experimental results were analyzed and tested using analysis of variance and non-parametric tests. The alpha diversity (Chao and Shannon indices) and the beta diversity (PERMANOVA) of the microbiome were calculated based on the OTU level. Tukey’s post hoc test of one-way analysis of variance (ANOVA) were performed in aluminum and *L. plantarum* levels of feces. A *P* value of less than 0.05 was used as a cutoff to indicate a statistically significant difference. The results were plotted using Origin 8.6 software (Originlab, Massachusetts, USA).

## Results

### Aluminum level in feces

Table 2 (Dataset S1) shows the weekly changes in the fecal aluminum content in tilapia. The fecal aluminum content was very low in the control and CCFM639 only groups, and was markedly elevated after aluminum exposure (*P* < 0.05). Moreover, the aluminum level in feces also increased as the duration of aluminum exposure increased. CCFM639 treatment promoted the elimination of aluminum in the feces. At the fourth week, the fecal aluminum level in the aluminum only group was 25.67 mg/kg. With the continuous administration of CCFM639, the fecal aluminum levels were significantly greater than those in the Al-only group at each test point (*P* < 0.05), up to 35.46 mg/kg at the third week.

**Table 2:** Effects of dietary supplementation with CCFM639 on aluminum contents in Nile tilapia feces.

Group	Aluminum level (mg/kg)				
	0 week	Week 1	Week 2	Week 3	Week 4
Control	1.13 ± 0.06^aA^	1.54 ± 0.07^aA^	1.83 ± 0.14^aA^	1.80 ± 0.10^aA^	1.54 ± 0.04^aA^
639 only	1.19 ± 0.04^aA^	1.55 ± 0.05^aA^	1.68 ± 0.13^aA^	1.74 ± 0.10^aA^	1.58 ± 0.06^aA^
Al only	1.08 ± 0.13^aA^	22.33 ± 1.31^bB^	23.67 ± 1.54^bB^	25.22 ± 1.32^bB^	25.67 ± 1.01^bB^
Al + 639	1.05 ± 0.08^aA^	25.67 ± 1.23^cB^	33.14 ± 2.53^cC^	35.46 ± 2.05^cC^	33.79 ± 2.37^cC^

**Notes.**

The data shown are the mean ± SEM for each group. The means with different superscript lowercase letters differ significantly among groups, and the superscript capital letters indicate a significant difference among time-points (*P* < 0.05).

### Quantification of Lactobacillus in feces

Table 3 (Dataset S2) shows that the amount of *L. plantarum* in tilapia feces in the CCFM639-only group was higher than control group by two orders of magnitude. By week 4, the content of *L. plantarum* in tilapia feces had been significantly decreased by aluminum exposure, from 10^5.4^ copies per gram of feces to 10^5.0^ copies per gram of feces (*P* < 0.05), whereas the addition of CCFM639 led to a significant increase in the *L. plantarum* content in feces to that in the 639-only group.

**Table 3:** Effects of dietary supplementation with CCFM639 on *L. plantarum* quantification in Nile tilapia feces.

Group	Number of *L. plantarum* (log copies/g feces)		
	Week 0	Week 2	Week 4
Control	5.49 ± 0.19^aA^	5.47 ± 0.09^aA^	5.40 ± 0.04^aA^
639 only	5.52 ± 0.01^aA^	7.83 ± 0.12^bB^	7.73 ± 0.02^bB^
Al only	5.49 ± 0.01^aA^	5.24 ± 0.08^aB^	4.99 ± 0.05^cC^
Al + 639	5.54 ± 0.13^aA^	7.46 ± 0.01^cB^	7.31 ± 0.13^dB^

**Notes.**

The data shown are the mean ± SEM for each group. The means with different superscript lowercase letters differ significantly among groups, and the superscript capital letters indicate a significant difference among time-points (*P* < 0.05).

### Intestinal microbial diversity and composition

**Figure 1:**
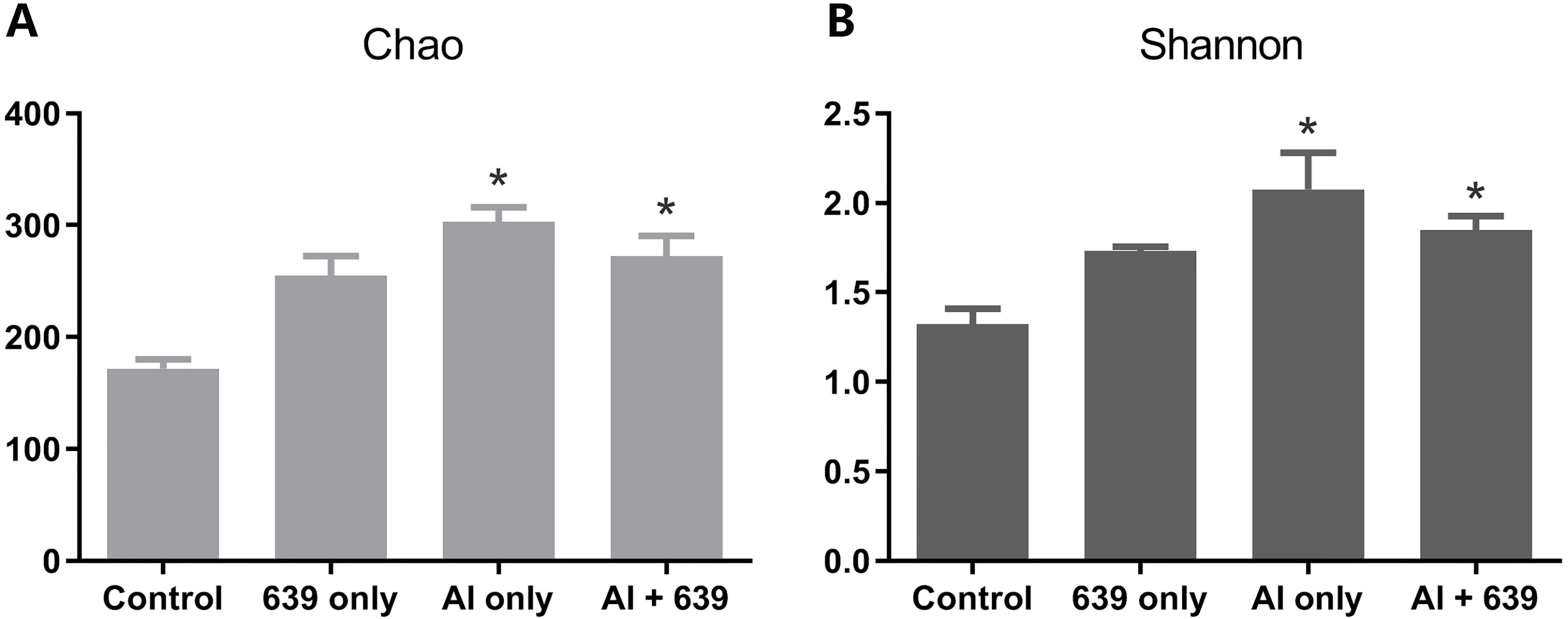
Alpha diversity results for the gut microbiota of Nile tilapia. The data shown are the means ± SEM for each group. Asterisks represent significant differences compared to the control group, *P* = 0.007 and 0.042 for Chao (A), *P* = 0.006 and 0.041 for Shannon (B).

**Figure 2:**
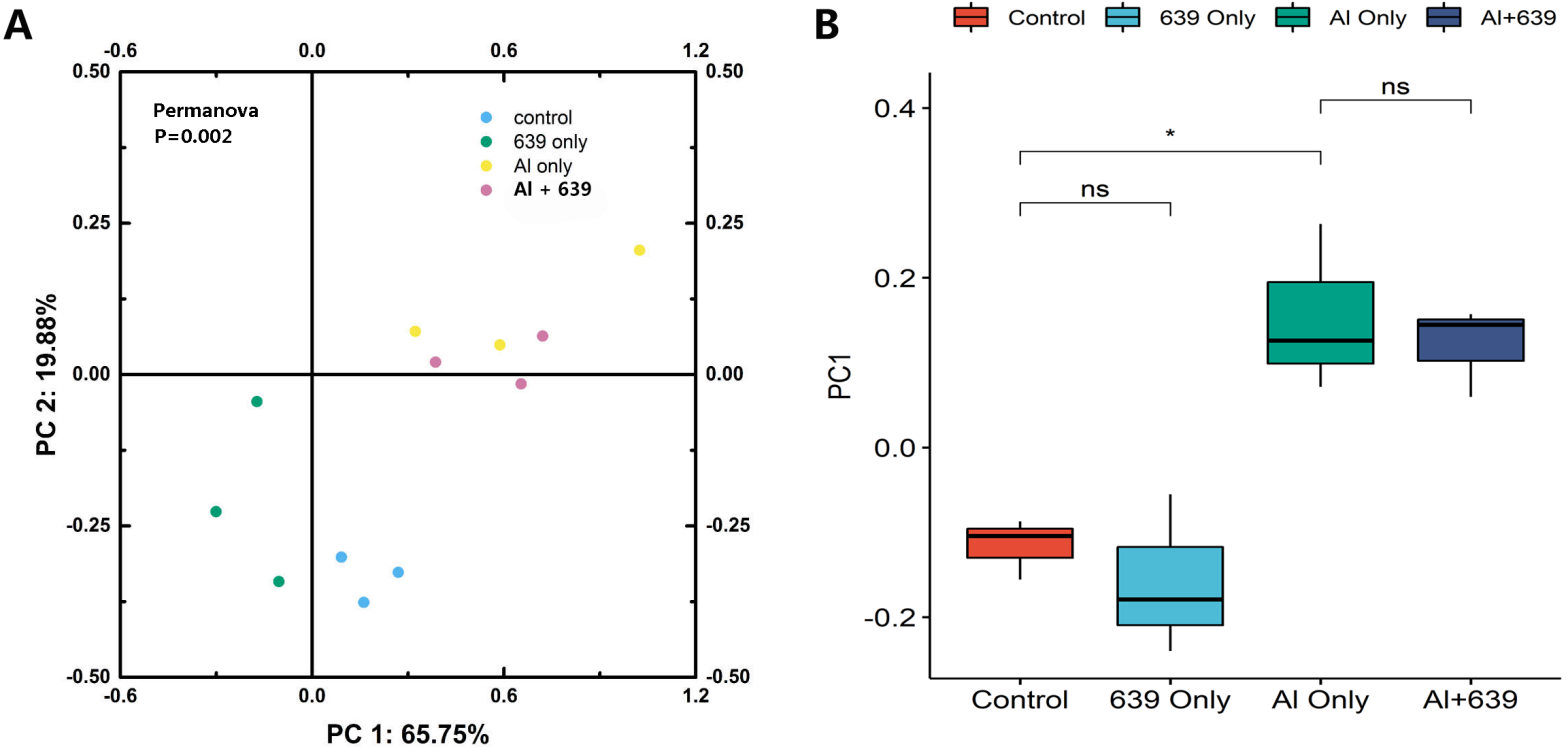
Principal coordinate score plots for the gut microbiota of Nile tilapia. (A) Unweighted unifrac distance. (B) PC1 values. The asterisk indicates the statistically significant differences ( *P* < 0.05) between different groups, ns indicates no statistically significant differences.

The numbers of OUTs of each group were 147.3, 172.0, 243.7, 206.3, respectively. Concerning alpha diversity microbiome analysis, Shannon and Chao1 indices were found higher in the 639 only and the Al+639 treatment groups compared to the control (Fig. 1, Dataset S3). As shown in Fig. 2A (Dataset S3), Principal Coordinate Analysis based on Unweighted unifrac distance indicated a overall significant clustering on the fecal microbiota composition (*P* = 0.002), in which PC1 explained 65.75% of the difference. Moreover, the results of PC1 analysis show that aluminum treatment had the greatest effect on the composition of the gut microbiota in tilapia (Fig. 2B, Dataset S3, *P* < 0.05).

**Figure 3:**
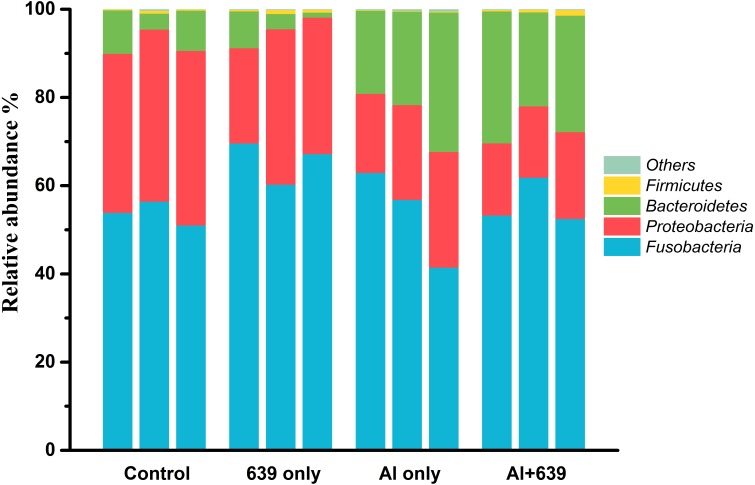
Effect of *L. plantarum* CCFM639 on the relative abundance (relative OTU composition) of the components of gut microbiota in Nile tilapia at the phylum level. .

Aluminum exposure changed the composition of the gut microbiota in Nile tilapia. The four predominant bacterial phyla Fusobacteria, Proteobacteria, Bacteroidetes, and Firmicutes, accounted for 53.74%, 38.21%, 7.54%, and 0.38%, respectively, in control group (Fig. 3, Dataset S4). Fish exposed to aluminum had a significantly greater abundance of Bacteroidetes and fewer Firmicutes, whereas administration of CCFM639 had the opposite effect. Interestingly, CCFM639 administration led to an increase in the abundance of Fusobacteria and Firmicutes and a decrease in the abundance of Proteobacteria.

**Figure 4:**
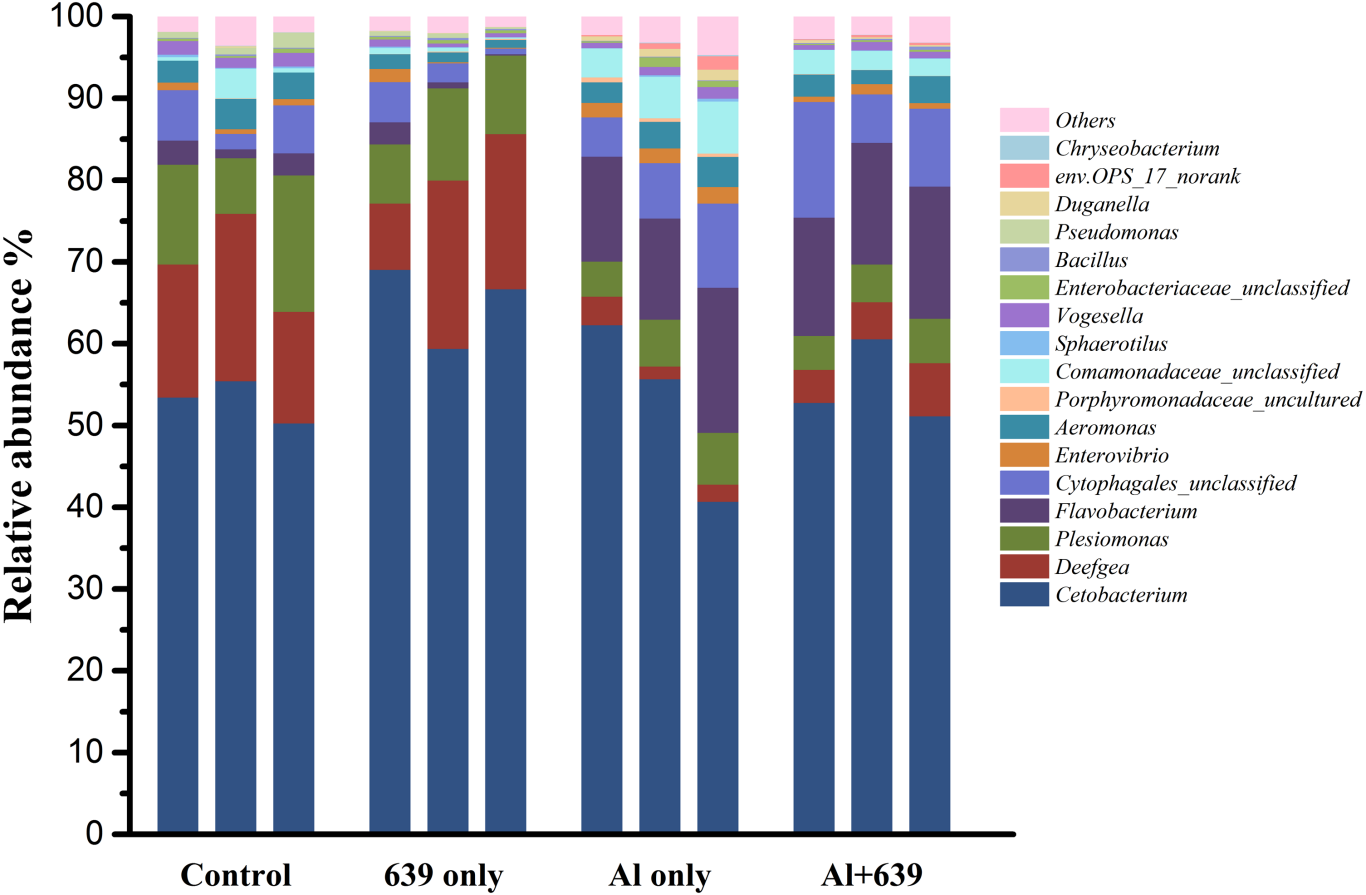
Effects of *L. plantarum* CCFM639 on the relative abundance (relative OTU composition) of the gut microbiota in Nile tilapia at the genus level.

**Figure 5:**
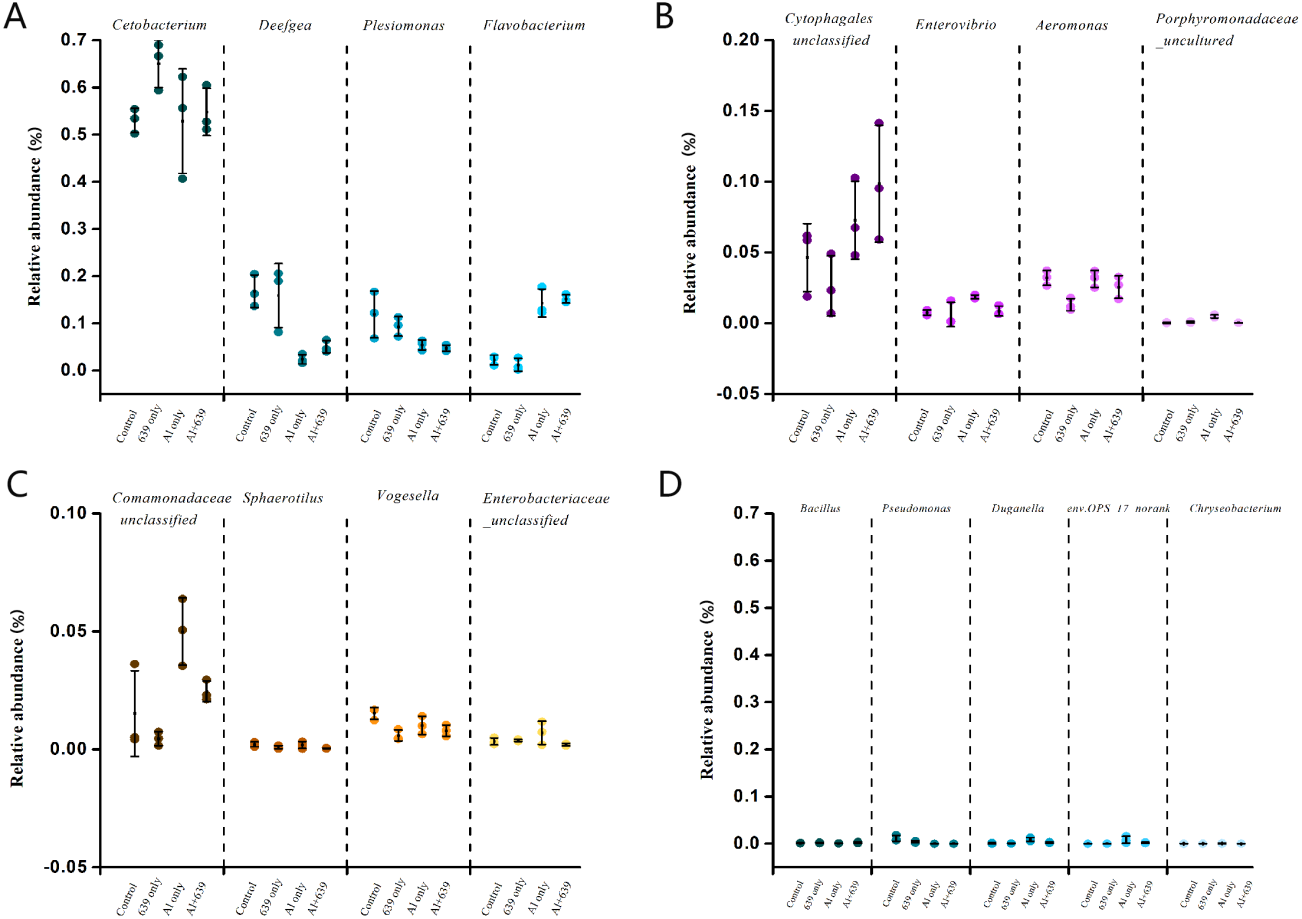
Relative abundance (relative OTU composition, % ± SEM) of the gut microbiota in Nile tilapia at the genus level. Relative abundance of Cetobacterium*,* Deefgea*,* Plesiomonas** and* Flavobacterium ;* (B)* Relative abundance of Cytophagales,* Enterovibrio*,* Aeromonas** and* Porphyromonadaceae;* (C)** Relative abundance of* Comamonadaceae,* Sphaerotilus*,* Vogesella** and* Enterobacteriaceae;* (D)** Relative abundance of** Bacillus*,* Pseudomonas*,* Duganella*, env.OPS_17-norank* and** Chryseobacterium. Data are expressed as mean ± SEM.

As shown in Fig. 4 (Dataset S5), 17 dominant genera were detected, the five most dominant were *Cetobacterium, Deefgea, Plesiomonas, Flavobacterium* and *Cytophagales*. Aluminum exposure significantly reduced the abundance of *Plesiomonas*, *Deefgea,* and *Pseudomonas* and drastically increased the abundance of *Flavobacterium*, *Enterovibrio*, and the families *Porphyromonadaceae* and *Comamonadaceae* (Fig. 5 and Table 4, Dataset S5; *P* < 0.05). In the tilapia exposed to aluminum, the administration of *L. plantarum* CCFM639 further led to a decrease in the abundance of *Enterovibrio, Comamonadaceae* and *Porphyromonadaceae* (*P* < 0.05). Unlike the results in the control group, supplementation with *L. plantarum* CCFM639 greatly reduced the abundance of *Aeromonas* and *Pseudomonas* (*P* < 0.05).

**Table 4:** Effects of CCFM639 on aluminum-induced changes in relative abundance of *Aeromonas, Enterovibrio, Comamonadaceae* and *Porphyromonadaceae* of Nile tilapia.

Group	*Aeromonas*	*Comamonadaceae*	*Enterovibrio*	*Porphyromonadaceae*
Control	3.20 ± 0.31^a^	1.52 ± 1.05^a^	0.76 ± 0.10^a^	0.03 ± 0.02^a^
639 only	1.31 ± 0.25^b^	0.45 ± 0.17^a^	0.62 ± 0.50^a^	0.09 ± 0.03^a^
Al only	3.14 ± 0.34^a^	5.00 ± 0.82^b^	1.86 ± 0.08^b^	0.49 ± 0.06^b^
Al + 639	2.57 ± 0.50^a^	2.46 ± 0.25^a^	0.87 ± 0.20^a^	0.04 ± 0.004^a^

**Notes.**

The data shown are the means ± SEM for each group. The different superscript letters represent significant differences between groups (*P* < 0.05).

## Discussion

Aquatic animals, especially fish, have direct contact with the water environment, which contains various pollutants and ever-changing microbiota (Egerton et al., 2018). An excessive aluminum concentration in the aquatic ecosystem can be harmful to the physiological functions of fish, and even threaten their survival (Egerton et al., 2018). In addition, the gut microbiota, which has a close association with fish health, can affect its metabolism, physiology, and immune function, and great inter-individual microbial diversity exists (Sullam et al., 2012). In this study, we adopted a culture-independent technology, next-generation sequencing, which is an up-to-date analytical method that can be widely used for analysis of host microbiota, including that of fish. More specifically, next-generation sequencing can be used to provide detailed information of low abundance microbiota can be provided and the genetic potential of species can even be predicted (Ghanbari, Kneifel & Domig, 2015). Our results show that the dominant four phyla in Nile tilapia were Fusobacteria, Proteobacteria, Bacteroidetes, and Firmicutes. Proteobacteria, Bacteroidetes, and Firmicutes accounted for 90% of the gut microbiota in a previous study of the teleost fishes (Giatsis et al., 2015; Liu et al., 2013; Ran et al., 2015). The intestinal microbiota of fish species including tilapia is characterized by various levels of Actinobacteria, Bacteroidetes, Fusobacteria, Proteobacteria and Firmicutes (Adeoye et al., 2016; Wu et al., 2013). At the genus level, the relative abundance of dominant *Cetobacterium* (affiliated with *Fusobacteria*) and *Plesiomonas* were also consistent with the results of a previous study in zebrafish (Ma et al., 2018).

**Figure 6:**
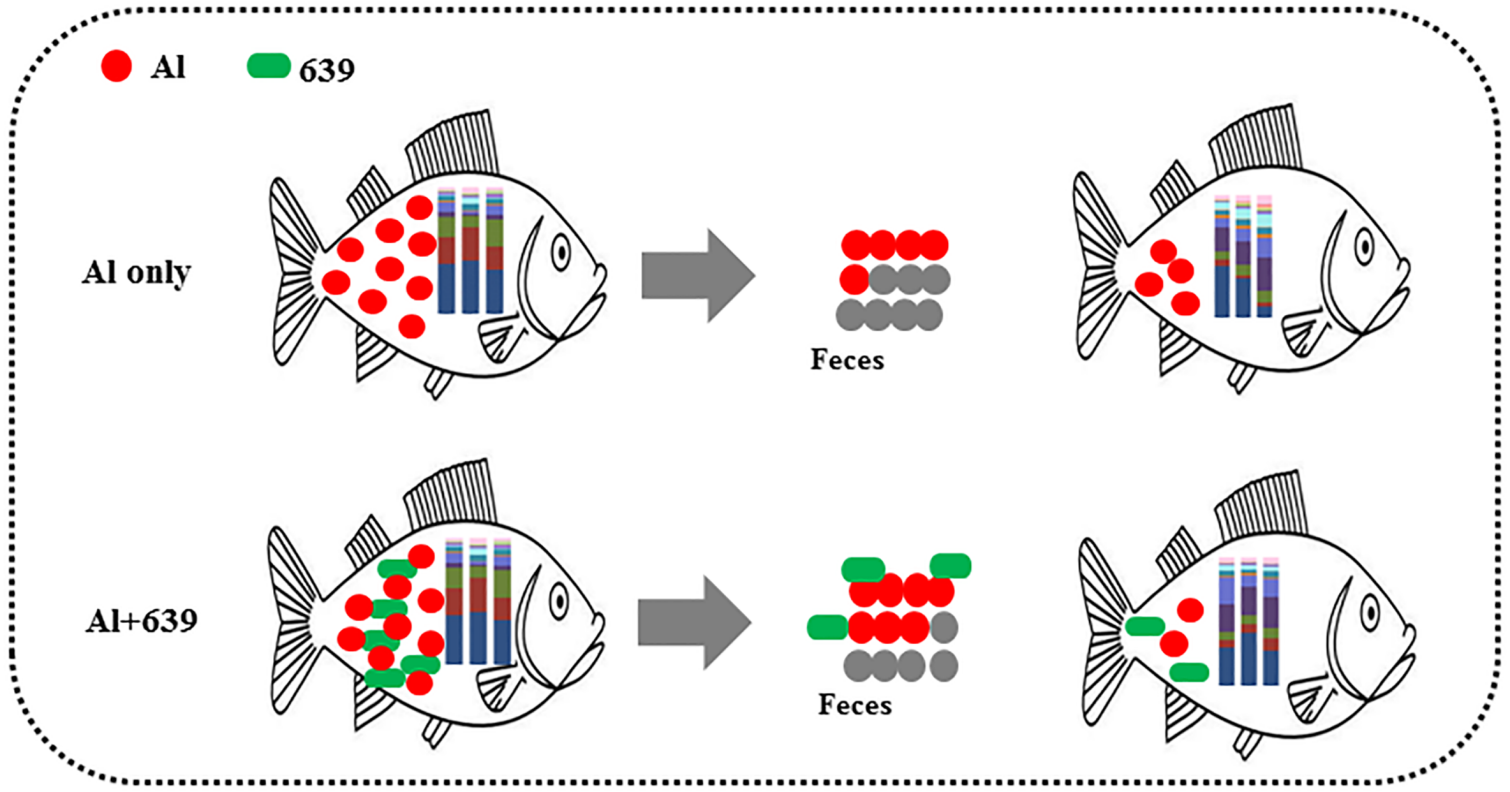
Potential protective mechanism of CCFM639 against aluminum induced gut injuries in Nile tilapia.

Despite various studies focusing on aluminum exposure and its effects on various host organs, including the liver, kidneys, and brain (Park et al., 2015), the link between aluminum exposure and the host intestinal microbiota remains unclear. The fish gut microbiota is commonly treated as an organ with a significant role in essential physiological functions and overall health. Consistent with our hypothesis, the fecal aluminum level of tilapia increased significantly when the fish were exposed to aluminum, and CCFM639 supplementation promoted aluminum excretion in feces as its excellent aluminum binding ability (Table 1, Fig. 6). These results are consistent with our previous study in a mouse model (Yu et al., 2016). In addition, the amount of *L. plantarum* in the tilapia feces increased markedly after the fish ingested feed mixed with a probiotic (Table 2). It is notable that aluminum exposure reduced the numbers of *L. plantarum* in tilapia feces at week 4 (Table 3), possibly due to a decrease in aluminum-intolerant *L. plantarum* in the gut caused by aluminum exposure.

Environmental exposure to aluminum altered the structure and relative abundance of the intestinal microbiota of Nile tilapia, resulting in a significant decrease in the relative abundance of the phyla Firmicutes and Proteobacteria and the genera *Deefgea*, *Plesiomonas* and *Pseudomonas* and elevated the relative abundance of the phylum Bacteroidetes, and the genera *Comamonadaceae* and *Porphyromonadaceae*, *Flavobacterium* and *Enterovibrio* (*P* < 0.05; Figs. 3, 4 and 5). The alpha diversity results show no significant difference between the control and 639 groups, and between the Al only and Al + 639 group. Accroding to previous studies (Qin et al., 2018; Uronis et al., 2011), we hypothesized that administration of a single probiotic CCFM639 may not exert a dramatic effect on the richness of the whole gut microbiota, but it may induce alterations in the abundance of specific genera. A previous study demonstrated that toxic metal exposure can influence the gut microbiota composition in mice (Zhai et al., 2017). Ingestion of aluminum damage the intestinal mucosa and reduce the intestinal barrier function and immune function (Vignal, Desreumaux & Body-Malapel, 2016). Pathogens and opportunistic pathogens in the intestinal tract can take the opportunity to multiply, causing disordered gut microbiota (Berg, 1996). A lower relative abundance of phylum Proteobacteria and a higher relative abundance of genus *Cetobacterium* were found after aluminum exposure. Exposure to silver led to a similar alteration in male zebrafish (Ma et al., 2018). The pathogen *Flavobacterium* is considered responsible for several fish diseases, including fry syndrome and bacterial cold water disease, which can cause high mortality levels in young fish (Leal et al., 2010; Nematollahi et al., 2003). The increase in the relative abundance of *Flavobacterium* may be due to its good aluminum tolerance capacity (more than 2,000 ppm) (Konishi et al., 1994). Similar speculation can be used to explain the increase in the relative abundance of *Comamonadaceae*. A series of metal-resistant genes and gene clusters was found in the whole-genome sequencing of the *Comamonas* strain, including arsenic, stibium, copper and so forth (Li et al., 2013; Lin et al., 2015). Wu et al., (2015) studied the virulence of several *Enterovibrio* and *Vibrio* strains for zebrafish; the results showed that *Enterovibrio* had a high level of virulence (LD_50_ values around 10^4^ CFU/g), and that *Vibrio* had a moderate level of virulence (LD_50_ values around 10^6^ CFU/g). *Enterovibrio* promotes the production of indole, which is a toxin that is harmful to intestinal lactic acid bacteria in excess quantities (Nowak & Libudzisz, 2006; Pascual et al., 2009). *Porphyromonadaceae* is a family in the of Bacteroidetes phylum that can also exert negative effects on the host (Russell et al., 2015). Therefore, aluminum exposure led to an increase in the relative abundance of some harmful bacteria. The dynamic changes in the gut microbiota can directly affect the intestinal mucosa and indirectly affect the health of the fish (Perez et al., 2010). The results help to further explain the harmful effects of aluminum exposure on the host’s growth and antioxidant system in previous study (Yu et al., 2017).

Probiotics have shown an excellent ability to resist disease and prevent pathogens (Hai, 2015). Probiotic supplementation results in improvements in microvilli density and length, which can increase the absorptive surface of the fish intestines and ultimately enhance the host’s physical barrier against potential pathogens (Standen et al., 2016). The use of probiotics stimulates the proliferation of a few probiotic bacteria and decreases the potential pathogens in fish. Numerous studies have demonstrated the significant effects of probiotics in protecting aquatic animals against infection by pathogens, such as the effects of *Bacillus* spp. against *Streptococcus iniae* (Cha et al., 2013), and the effects of *Pseudomonas* spp. against *F. psychrophilum* (Korkea-aho et al., 2012). Compared with the Al-only group, the administration of CCFM639 significantly reduced the abundance of *Enterovibrio, Comamonadaceae* and *Porphyromonadaceae*, indicating the potential protective effect of CCFM639 treatment against aluminum-induced increases in pathogen. *Aeromonas* is one of the fish pathogens that causes several fish diseases, including motile aeromonad septicemia, furunculosis, ulcer disease, and carp erythrodermatitis (Miller & Harbottle, 2018). In the 639 only group, the abundance of *Aeromonas* was decreased, which indicates modification of the bacterial community composition by administration of the probiotic CCFM639. Studies in tilapia have also demonstrated an improvement in gut microbiota with the addition of probiotics (Standen et al., 2015).

## Conclusions

In conclusion, the accumulated aluminum in Nile tilapia can be excreted through feces, and oral administration of *L. plantarum* CCFM639 increased aluminum excretion (Fig. 6). Moreover, environmental exposure to aluminum altered the composition of the gut microbiota, and alteration in the levels of *Enterovibrio, Comamonadaceae,* and *Porphyromonadaceae* can be recovered by administration of CCFM639. Therefore, this study provides a further explanation of the protective mechanisms of CCFM639 against aluminum toxicity. Apart from promotion of aluminum discharge through feces, regulation of the gut microbiota with *L. plantarum* CCFM639 may be an underlying mechanism by which aluminum toxicity is alleviated.

##  Supplemental Information

10.7717/peerj.6963/supp-1 Dataset S1 The raw data of Al contents in Nile tilapia feces

10.7717/peerj.6963/supp-2 Dataset S2 The raw data of *L. plantarum* quantification in Nile tilapia feces

10.7717/peerj.6963/supp-3 Dataset S3 The raw data of alpha diversity for the gut microbiota of Nile tilapia

10.7717/peerj.6963/supp-4 Dataset S4 The raw data of the relative abundance of gut microbiota in Nile tilapia at the phylum level

10.7717/peerj.6963/supp-5 Dataset S5 The raw data of the relative abundance of gut microbiota in Nile tilapia at the genus level

## Additional Information and Declarations

### Data Availability

The following information was supplied regarding data availability: The raw measurements are available in the Supplemental Files.
