# Supplementary material for: Dietary supplementation with probiotics regulates gut microbiota structure and function in Nile tilapia exposed to aluminum
Source: PeerJ. 2019 Jun 3;7:e6963. doi: 10.7717/peerj.6963 (PMC6553448; doi:10.7717/peerj.6963)
Supplement: Dataset S1 [file peerj-07-6963-s001.docx]

| **Al levels in feces** | | | | | |
| --- | --- | --- | --- | --- | --- |
|  | **0 week** | **Week 1** | **Week 2** | **Week 3** | **Week 4** |
| **Control** | 1.02 | 1.63 | 2.00 | 1.97 | 1.51 |
|  | 1.22 | 1.41 | 1.56 | 1.64 | 1.50 |
|  | 1.14 | 1.59 | 1.92 | 1.79 | 1.62 |
| **639 only** | 1.10 | 1.66 | 1.93 | 1.94 | 1.69 |
|  | 1.24 | 1.49 | 1.47 | 1.59 | 1.47 |
|  | 1.22 | 1.51 | 1.64 | 1.69 | 1.58 |
| **Al only** | 0.90 | 20.00 | 21.00 | 28.00 | 28.00 |
|  | 1.34 | 25.00 | 27.00 | 23.67 | 24.00 |
|  | 0.99 | 22.00 | 23.00 | 24.00 | 25.00 |
| **Al+639** | 0.90 | 28.00 | 38.15 | 32.04 | 30.11 |
|  | 1.12 | 24.00 | 30.03 | 39.12 | 38.21 |
|  | 1.13 | 25.00 | 31.23 | 35.21 | 33.04 |
