# Supplementary material for: Dietary supplementation with probiotics regulates gut microbiota structure and function in Nile tilapia exposed to aluminum
Source: PeerJ. 2019 Jun 3;7:e6963. doi: 10.7717/peerj.6963 (PMC6553448; doi:10.7717/peerj.6963)
Supplement: Dataset S2 [file peerj-07-6963-s002.docx]

| **Number of *L. plantarum*（log copies/g feces）** | | | |
| --- | --- | --- | --- |
|  | **Week0** | **Week 2** | **Week 4** |
| **Control** | 5.34 | 5.62 | 5.32 |
|  | 5.27 | 5.32 | 5.49 |
|  | 5.86 | 5.47 | 5.38 |
| **639 only** | 5.53 | 7.95 | 7.77 |
|  | 5.54 | 7.96 | 7.72 |
|  | 5.51 | 7.58 | 7.71 |
| **Al only** | 5.51 | 5.12 | 5.09 |
|  | 5.47 | 5.19 | 4.90 |
|  | 5.49 | 5.40 | 4.99 |
| **Al+639** | 5.50 | 7.47 | 7.43 |
|  | 5.35 | 7.46 | 7.45 |
|  | 5.78 | 7.44 | 7.05 |
