# Supplementary material for: Dietary supplementation with probiotics regulates gut microbiota structure and function in Nile tilapia exposed to aluminum
Source: PeerJ. 2019 Jun 3;7:e6963. doi: 10.7717/peerj.6963 (PMC6553448; doi:10.7717/peerj.6963)
Supplement: Dataset S3 [file peerj-07-6963-s003.docx]

| **Alpha diversity results for the gut microbiota of Nile tilapia** | | | | |
| --- | --- | --- | --- | --- |
| **Group** | **Reads** | **0.97** | | |
|  |  | **OTU** | **chao** | **shannon** |
| F35_1 | 75849 | 140 | 159 | 1.69 |
| F35_2 | 92928 | 167 | 188 | 1.72 |
| F35_3 | 86729 | 135 | 168 | 1.78 |
| F36_1 | 68923 | 181 | 285 | 1.41 |
| F36_2 | 77571 | 168 | 225 | 1.41 |
| F36_3 | 89077 | 167 | 256 | 1.15 |
| F37_1 | 75697 | 207 | 281 | 1.74 |
| F37_2 | 75645 | 261 | 325 | 2.05 |
| F37_3 | 74265 | 263 | 304 | 2.44 |
| F38_1 | 74122 | 212 | 290 | 1.87 |
| F38_2 | 84737 | 210 | 291 | 1.7 |
| F38_3 | 71133 | 197 | 235 | 1.97 |
