# Supplementary material for: Dietary supplementation with probiotics regulates gut microbiota structure and function in Nile tilapia exposed to aluminum
Source: PeerJ. 2019 Jun 3;7:e6963. doi: 10.7717/peerj.6963 (PMC6553448; doi:10.7717/peerj.6963)
Supplement: Dataset S4 [file peerj-07-6963-s004.docx]

| **The relative abundance of the components of gut microbiota at the phylum level** | | | | | |
| --- | --- | --- | --- | --- | --- |
|  | **Fusobacteria** | **Proteobacteria** | **Bacteroidetes** | **Firmicutes** | **Others** |
| **Control** | 53.86 | 36.04 | 9.85 | 0.21 | 0.04 |
|  | 56.38 | 39.00 | 3.62 | 0.64 | 0.35 |
|  | 50.98 | 39.58 | 9.13 | 0.28 | 0.02 |
| **639 only** | 69.56 | 21.62 | 8.35 | 0.32 | 0.15 |
|  | 60.24 | 35.23 | 3.44 | 0.93 | 0.16 |
|  | 67.16 | 30.94 | 1.15 | 0.63 | 0.12 |
| **Al only** | 62.91 | 17.91 | 18.85 | 0.15 | 0.19 |
|  | 56.78 | 21.49 | 21.19 | 0.19 | 0.36 |
|  | 41.43 | 26.21 | 31.63 | 0.21 | 0.51 |
| **Al+639** | 53.25 | 16.33 | 29.92 | 0.35 | 0.14 |
|  | 61.82 | 16.18 | 21.29 | 0.60 | 0.11 |
|  | 52.47 | 19.67 | 26.43 | 1.26 | 0.17 |
