# Supplementary material for: Dietary supplementation with probiotics regulates gut microbiota structure and function in Nile tilapia exposed to aluminum
Source: PeerJ. 2019 Jun 3;7:e6963. doi: 10.7717/peerj.6963 (PMC6553448; doi:10.7717/peerj.6963)
Supplement: Dataset S5 [file peerj-07-6963-s005.docx]

| **The relative abundance of the components of gut microbiota at the genus level** | | | | | | | | | | | | | | | | | | |
| --- | --- | --- | --- | --- | --- | --- | --- | --- | --- | --- | --- | --- | --- | --- | --- | --- | --- | --- |
|  | **Cetobacterium** | **Deefgea** | **Plesiomonas** | **Flavobacterium** | **Cytophagales_unclassified** | **Enterovibrio** | **Aeromonas** | **Porphyromonadaceae_uncultured** | **Comamonadaceae_unclassified** | **Sphaerotilus** | **Vogesella** | **Enterobacteriaceae_unclassified** | **Bacillus** | **Pseudomonas** | **Duganella** | **env.OPS_17_norank** | **Chryseobacterium** | **Others** |
| **Control** | 53.426 | 16.273 | 12.195 | 2.946 | 6.188 | 0.929 | 2.654 | 0.009 | 0.427 | 0.308 | 1.675 | 0.287 | 0.086 | 0.727 | 0.010 | 0.001 | 0.001 | 1.857 |
|  | 55.407 | 20.475 | 6.803 | 1.092 | 1.884 | 0.568 | 3.720 | 0.070 | 3.618 | 0.109 | 1.238 | 0.225 | 0.183 | 0.822 | 0.192 | 0.006 | 0.039 | 3.551 |
|  | 50.251 | 13.663 | 16.684 | 2.705 | 5.858 | 0.771 | 3.238 | 0.010 | 0.515 | 0.215 | 1.677 | 0.501 | 0.122 | 1.845 | 0.010 | 0.007 | 0.004 | 1.924 |
| **639 only** | 69.033 | 8.127 | 7.221 | 2.715 | 4.911 | 1.611 | 1.790 | 0.025 | 0.746 | 0.166 | 0.857 | 0.346 | 0.099 | 0.526 | 0.080 | 0.013 | 0.013 | 1.722 |
|  | 59.389 | 20.565 | 11.291 | 0.720 | 2.330 | 0.139 | 1.189 | 0.113 | 0.462 | 0.077 | 0.444 | 0.425 | 0.257 | 0.552 | 0.051 | 0.006 | 0.003 | 1.988 |
|  | 66.680 | 18.971 | 9.602 | 0.155 | 0.697 | 0.106 | 0.958 | 0.135 | 0.161 | 0.028 | 0.476 | 0.363 | 0.171 | 0.203 | 0.038 | 0.000 | 0.001 | 1.254 |
| **Al only** | 62.279 | 3.496 | 4.264 | 12.841 | 4.816 | 1.755 | 2.512 | 0.614 | 3.541 | 0.030 | 0.650 | 0.192 | 0.062 | 0.000 | 0.536 | 0.166 | 0.004 | 2.242 |
|  | 55.659 | 1.568 | 5.739 | 12.365 | 6.754 | 1.818 | 3.232 | 0.450 | 5.067 | 0.197 | 1.000 | 1.176 | 0.096 | 0.003 | 0.939 | 0.694 | 0.068 | 3.175 |
|  | 40.685 | 2.095 | 6.340 | 17.751 | 10.273 | 2.014 | 3.689 | 0.411 | 6.381 | 0.330 | 1.420 | 0.739 | 0.096 | 0.003 | 1.311 | 1.635 | 0.122 | 4.707 |
| **Al+639** | 52.763 | 4.043 | 4.146 | 14.483 | 14.149 | 0.648 | 2.709 | 0.030 | 2.956 | 0.033 | 0.562 | 0.151 | 0.112 | 0.001 | 0.359 | 0.093 | 0.023 | 2.738 |
|  | 60.540 | 4.546 | 4.612 | 14.875 | 5.922 | 1.267 | 1.707 | 0.044 | 2.304 | 0.064 | 1.039 | 0.221 | 0.166 | 0.000 | 0.173 | 0.287 | 0.016 | 2.217 |
|  | 51.141 | 6.481 | 5.441 | 16.153 | 9.538 | 0.690 | 3.281 | 0.033 | 2.123 | 0.042 | 0.800 | 0.232 | 0.399 | 0.001 | 0.179 | 0.271 | 0.026 | 3.168 |
